# Novel Therapeutic Approaches in the Management of Chronic Kidney Disease

**DOI:** 10.3390/biomedicines11102746

**Published:** 2023-10-11

**Authors:** Bartłomiej Dąbek, Jill Dybiec, Weronika Frąk, Piotr Fularski, Wiktoria Lisińska, Ewa Radzioch, Ewelina Młynarska, Jacek Rysz, Beata Franczyk

**Affiliations:** 1 Department of Nephrocardiology, Medical University of Lodz, ul. Zeromskiego 113, 90-549 Lodz, Poland; 2 Department of Nephrology, Hypertension and Family Medicine, Medical University of Lodz, ul. Zeromskiego 113, 90-549 Lodz, Poland

**Keywords:** chronic kidney disease, kidney failure, renal insufficiency, renal replacement therapy, kidney transplantation, renal diet, finerenone, canakinumab

## Abstract

Chronic kidney disease (CKD) is a progressive and incurable disease that impairs kidney function. Its prevalence is estimated to affect up to 800 million individuals within the general population, and patients with diabetes and hypertension are particularly at risk. This disorder disrupts the physiological mechanisms of the body, including water and electrolyte balance, blood pressure regulation, the excretion of toxins, and vitamin D metabolism. Consequently, patients are exposed to risks such as hyperkalemia, hyperphosphatemia, metabolic acidosis, and blood pressure abnormalities. These risks can be reduced by implementing appropriate diagnostic methods, followed by non-pharmacological (such as physical activity, dietary, and lifestyle adjustment) and pharmacological strategies after diagnosis. Selecting the appropriate diet and suitable pharmacological treatment is imperative in maintaining kidney function as long as possible. Drugs such as finerenone, canakinumab, and pentoxifylline hold promise for improved outcomes among CKD patients. When these interventions prove insufficient, renal replacement therapy becomes essential. This is particularly critical in preserving residual renal function while awaiting renal transplantation or for patients deemed ineligible for such a procedure. The aim of this study is to present the current state of knowledge and recent advances, providing novel insights into the treatment of chronic kidney disease.

## 1. Introduction

**Figure 1:**
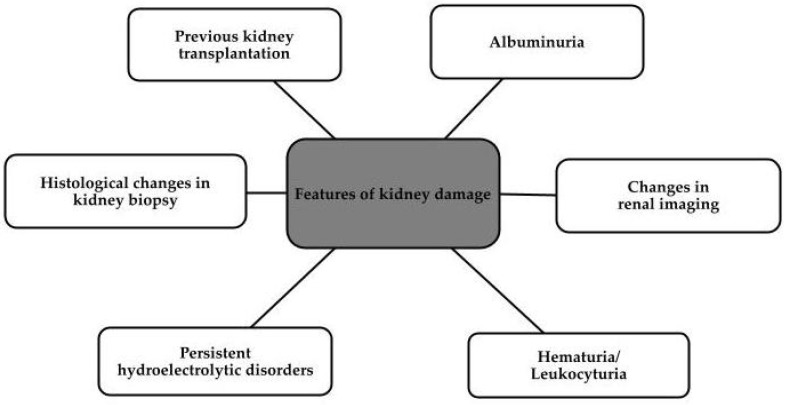
Features of kidney damage defining chronic kidney disease [1].

Chronic kidney disease (CKD) affects about 800 million people worldwide [1], including more than 20 million Americans [2]. It is defined as a pathology that is a process leading to changes in the structure and/or function of the kidneys. It is characterized by both an inability to reverse the changes that occur and a slow progression [1,3]. Patients with this disease have a glomerular filtration rate (GFR) of less than 60 mL/min/1.73 m^2^ for 3 months or more, or a GFR greater than 60 mL/min/1.73 m^2^, but with specific features of kidney damage, which are shown in Figure 1 below [1].

Many papers show that a decrease in GFR and the presence of albuminuria can cause increased cardiovascular risk and mortality from any cause, hence the importance of early diagnosis [4]. According to some data from 2013, a decrease in GFR was associated with the death of about 2.2 million people [1].

As for the classification of chronic kidney disease, there are grades from I-V related to the value of GFR [1]. A more detailed description is presented in Table 1 [5].

**Figure 2:**
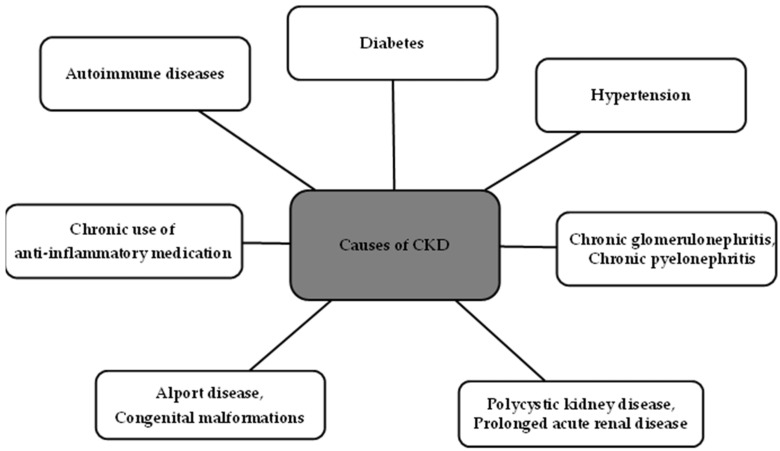
Causes of chronic kidney disease [1].

The causes of chronic kidney disease are various, and the most important ones are indicated in Figure 2 below [1].

The purpose of this paper is to present the diagnosis, the latest therapy, both pharmacological and non-pharmacological, looking at dialysis and transplantation.

## 2. Diagnosis

CKD is usually discovered as a result of routine screening with a blood chemical profile and urine tests, or as an incidental finding [6]. Patients with symptoms such as gross hematuria, “foamy urine” (an indication of albuminuria), nocturia, flank discomfort, or reduced urine production are less prevalent. Patients with advanced CKD may have tiredness, low appetite, nausea, vomiting, metallic taste, unintended weight loss, pruritus, mental state changes, dyspnea, or peripheral edema [7]. When evaluating a patient with known or suspected CKD, clinicians should ask about any additional symptoms that may indicate a systemic cause (e.g., hemoptysis, rash, lymphadenopathy, hearing loss, neuropathy) or urinary obstruction (e.g., urinary hesitancy, urgency, frequency, or incomplete bladder emptying). Furthermore, patients should be evaluated for risk factors for kidney disease; such as past exposure to possible nephrotoxins (e.g., nonsteroidal anti-inflammatory drugs (NSAIDs), phosphate-based bowel preparations, herbal remedies containing aristolochic acid, antibiotic therapies such as gentamicin, and chemotherapies), a history of nephrolithiasis or recurrent urinary tract infections, the presence of comorbidities (e.g., hypertension, diabetes, autoimmune disease, chronic infections), and a family history of kidney disease; and, if available, a thorough physical examination, which should include a meticulous evaluation of a patient’s volume status, may give further insights about the underlying etiology of CKD [8]. Volume depletion symptoms can be caused by low oral intake, vomiting, diarrhea, or overdiuresis, whereas volume overload symptoms can be caused by decompensated heart failure, liver failure, or nephrotic syndrome. On ocular examination, the appearance of arterial–venous nicking or retinopathy implies long-standing hypertension or diabetes. Renovascular disease may be present in patients who have carotid or abdominal bruits. If you have flank discomfort or enlarged kidneys, you should be evaluated for obstructive uropathy, nephrolithiasis, pyelonephritis, or polycystic kidney disease. Diabetes can cause neuropathy, as can vasculitis or amyloidosis. Rashes (systemic lupus erythematosus, acute interstitial nephritis), palpable purpura (Henoch–Schonlein purpura, cryoglobulinemia, vasculitis), and telangiectasias are all possible skin abnormalities such as vasculitis and telangiectasias [6]. CKD is defined as a chronic impairment in kidney structure or function (e.g., GFR 60 mL/min/1.73 m^2^ or albuminuria 30 mg per 24 h) for more than 3 months. Diabetes and hypertension are the most frequent causes of CKD in affluent nations. The interplay between hypertension and CKD is complicated, increasing the risk of poor cardiovascular and cerebrovascular consequences. This is especially important in the case of resistant hypertension, which is frequent in CKD patients. The pathophysiology of CKD-related hypertension is complex, with several pathways contributing to hypertension. These pathogenic processes include sodium dysregulation, increased sympathetic nervous system activity, and changes in the function of the renin–angiotensin–aldosterone system [9,10]. The control of Na+ and water is complicated, including neuro-humeral multi-organ mechanisms such as glucocorticoids, RAS (renin–angiotensin system) activation, muscle catabolism, urea production, and salt retention. Evidence shows that the general population’s excessive salt and poor water intake leads to hypertension and CKD. The non-osmotic Na+ storage pool in the skin/muscle most likely contributes to Na^+^ and water homeostasis. Even little increases in water and salt intake can help to reduce arginine vasopressin (AVP) and blood pressure rises. Preventive education for the general public is both cost-effective and helpful [11] when it comes to how renal failure alters the amount, content, and quality of blood lipids, favoring an atherogenic profile. Patients with severe CKD or end-stage renal disease (ESRD) have a distinct lipid pattern characterized by hypertriglyceridemia and low HDL cholesterol levels but normal LDL cholesterol levels. There is a definite link between LDL cholesterol and major atherosclerotic events in the general population. LDL cholesterol, on the other hand, has a negative correlation with these outcomes in individuals with ESRD at lower LDL cholesterol levels and a flat or moderately positive association with mortality at higher LDL cholesterol levels [12]. Staging and novel risk assessment techniques that combine GFR and albuminuria in CKD patients can assist guide therapy, monitoring, and referral strategies. Optimal CKD care includes lowering cardiovascular risk (e.g., statins and blood pressure control), treating albuminuria (e.g., angiotensin-converting enzyme inhibitors or angiotensin II receptor blockers), and avoiding possible nephrotoxins (e.g., NSAIDs) [13].

## 3. Therapies

Treatment is very broad and involves many different ways to prevent, treat, and slow CKD. The most important goals are slowing the progression of CKD; treating pathology-related complications such as anemia, mineral and bone disorder, hydroelectrolytic disorders, metabolic acidosis, and cardiovascular disease; preparing the patient for kidney replacement therapy (KRT), and establishing an immunization routine, particularly for hepatitis B. It is critical to emphasize that a multidisciplinary team, notably of nutrition, nursing, psychiatry, and social aid, is required at all levels of therapy [1].

### 3.1. Non-Pharmacological Therapies

There are a few non-pharmacological methods that can be implemented in the CKD patient group, in order to decelerate kidney function decline, that at the same time can lead to postponement in time, or complete avoidance of dialysis therapy in future. To achieve these goals, methods like raising the amount of physical activity (PA), abstaining from smoking, specific diets including sodium-restricted diet, and losing excess weight are being implemented [14]. One of the main non-pharmacological therapies with a range of health benefits is increasing physical activity. This one among others positively affects the blood pressure (BP), lipid profile, and the level of inflammatory biomarkers, which delays CKD progression, but this is just the beginning of the health advantages derived from engaging in PA [15]. Exercising is also shown to reduce insulin resistance (IR), which plays a pivotal role in the pathophysiology of T2DM (type 2 diabetes mellitus), which is a disease that can lead to diabetic nephropathy and by that to CKD progression [16,17]. What is more, PA leads to an improvement in quality of life in this patient group, but it may even cause slight elevation of eGFR (estimated glomerular filtration rate), amounting to around (+2.6 mL/min) [18].

Another, but also significant non-pharmacological method of CKD therapy is incorporation of proper diet (especially noteworthy is plant-based diet). The fact that conventional dietary guidelines for patients suffering from CKD focus mainly on the amount of consumed nutrients may result in a reduced consumption of vegetables and fruits. This can lead to the loss of the benefits derived from consumption of higher amounts of fiber which are, among others, shifting the gut microbiota to reduce production of uremic toxins; it also implies lower intake of plant fats, like olive oil which shows anti-atherogenic properties or plant anions, that could alleviate metabolic acidosis and decelerate CKD development [19]. Despite the fact that a plant-based diet can result in hyperkaliemia, more and more evidence supports various health advantages of a plant-based diet in the mentioned patient group. This includes improvement of blood pressure, a reduced risk of CKD advancement, and, what is more, a reduced risk of CVD (cardiovascular disease) and stroke occurrence [20]. When it comes down to the diet, patients suffering from CKD should avoid a high-protein diet, which is understood as the consumption of more than 1.2 g per kilogram of body mass per day. Over time, this may result in kidney dysfunction, due to the induction and long-term conduction of glomerular hyperfiltration [21]. The next important factor in terms of diet in mentioned disease is proper sodium intake. Elevated sodium consumption in chronic kidney disease inevitably induces higher blood pressure among the other effects, by stimulating the central nervous system (CNS), causing fluid overload or increasing the intrarenal production of angiotensin-II. Moreover, increased salt intake implies synthesis stimulation of pro-inflammatory cytokines and enhanced oxidative stress. According to that, a low-sodium diet is helpful when it comes down to controlling hypertension, regardless of BP level. It is especially important due to the fact that elevated BP is one of the causes of CKD progression [22,23].

Last but not least, a way to improve health in CKD is avoiding usage of substances such as alcohol and cigarettes, especially in higher amounts. Both significant alcohol abuse and smoking by itself are linked with elevated risk of declining kidney function [24].

### 3.2. Dietary Patterns

The mainstay for the management of CKD patients comprises pharmacological and nutritional therapies. There is a growing body of evidence of the role of diet in the treatment of kidney diseases and their ensuing complications [25]. Proper nutrition can help slow the progression of CKD, manage symptoms, and prevent complications. Furthermore, the quality of diet (higher intake of vegetables, fiber, and whole grains) could have an impact on the production of uremic toxins and the diversity, composition, and functioning of the gut microbiota in adults affected by CKD [26]. The recommended dietary allowances for individuals with CKD advise adhering to a low protein intake (ranging from 0.3 to 0.8 g per kilogram of body weight per day), restricting sodium to less than 2.3 g per day, limiting phosphate intake to below 700 milligrams per day, and considering supplementation of essential amino acids, ketoacids, calcium carbonate, vitamins, and iron [27]. The kidney plays a crucial role in phosphate homeostasis, therefore progression of CKD leads to hyperphosphatemia. It is crucial to deliver nutritional counseling for hyperphosphatemia in patients with CKD. Valente et al. demonstrated the effectiveness of dietary advice in the reduction in serum phosphorus levels [28]. Furthermore, pharmacological management is required to control hyperphosphatemia, such as phosphate binders (e.g., sevelamer carbonate) [29,30,31].

Ketogenic diets (KD) are a popular and frequently used diet for weight loss. A ketogenic diet is a high-fat, low-carbohydrate dietary approach that shifts the body into a state of ketosis, where it primarily relies on fats and ketones for energy, instead of carbohydrates [32]. It is suggested that KD could be considered a new strategy for managing and treating CKD [33]. Bruci et al. conducted a nonrandomized, prospective study involving 92 participants (38 with stage 2 chronic kidney disease and 54 with normal kidney function), who followed a very-low-calorie KD (450–800 kcal/day) for about 3 months; there was no significant difference in serum creatinine or eGFR. However, when focusing on participants with an initial eGFR of 60–89 mL/min/1.73 m^2^, a separate analysis revealed a statistically significant increase in eGFR from 76.32 to 82.21 mL/min/1.73 m^2^ [34]. In addition, KD might have the potential to manage polycystic kidney disease (PKD) due to its ability to inhibit cyst growth in animal models. Although clinical research involving humans is still being conducted, Torres et al. evaluated 20 individuals affected by autosomal dominant PKD and an eGFR between 24 and 94 mL/min/1.73 m^2^. The study focused on a plant-centered KD and observed positive outcomes, such as a mean reduction of 5.6% in body weight, a 16.5% decrease in fasting blood glucose levels, and an average eGFR increase of 8.6% [35]. Nevertheless, these dietary approaches have the potential to cause health risks. Observational research has linked the intake of saturated and animal fats, which are prominent components of conventional KD, with higher albuminuria [36,37]. Furthermore, KD is associated with an increased risk of dyslipidemia and hyperlipidemia, as well as its link to increased mortality risks, according to observational studies [38,39]. What is worth noting, it is advised to obtain carbohydrates and fats from whole, unprocessed, fiber-packed plant-based sources. Notably, a low-carbohydrate diet centered around plant foods has demonstrated weight loss benefits and enhanced cardiovascular risk factors in individuals without CKD [40,41].

Limited clinical trials have been conducted on the effects of intermittent fasting (IF) on CKD. However, Malik et al. investigated the effects of IF on patients with CKD during Ramadan. Nevertheless, these studies have found either an improvement, a decline, or no alteration in kidney function [42]. Restriction from water and medications during fasting hours in Ramadan potentially influences the results. Additionally, the lack of controlled conditions in these studies might contribute to the inconsistent outcomes. However, when considering the proper allowance of water and medications, IF could have the potential to offer significant health advantages, but additional research is essential.

Another dietary pattern reviewed for patients with kidney diseases is plant-based diet (PBD). It prioritizes the intake of plant-derived foods and may incorporate limited quantities of meat, fish, seafood, eggs, and dairy products, depending on individual preferences and choices [43]. This type of diet offers numerous potential advantages for both preventing and managing CKD [44]. Carrero et al. established that PBD has promising potential for individuals with CKD [19]. First of all, higher fiber consumption can alter gut microbiota to produce fewer uremic toxins. Further, plant-based fats, notably olive oil, exhibit anti-atherogenic properties. Plant-derived anions could help alleviate metabolic acidosis and potentially slow CKD progression. Due to the lower bioavailability of phosphorus in plants compared to animal sources, PBD might better manage hyperphosphatemia [19,45,46]. Salomo et al. also found that implementing a short-term PBD has beneficial effects on phosphorus homeostasis among individuals with CKD in Stages 3 and 4 [47]. Recently, Hansen et al. investigated the impact of PBD on metabolic acidosis and uremic toxins in 18 patients with moderate CKD, specifically in stages 3–4 of the disease. A one-week trial of PBD among these patients demonstrated significant improvements in metabolic acidosis. However, these findings need future research on the wide-ranging impact of PBD on kidney preservation in CKD [48].

The Mediterranean diet appears to be associated with the maintenance of kidney function, improved cardiometabolic profile and lowered risk of mortality in individuals diagnosed with CKD [49,50]. A meta-analysis of studies involving the general population revealed that for each additional point of adherence to the Mediterranean Diet Scale, there was a 10% reduction in the odds of developing CKD [51]. Research conducted on individuals diagnosed with CKD also indicates positive results from adopting a Mediterranean dietary pattern. These results include improved cardiometabolic indicators, reduced risk of developing kidney failure and lower mortality rates. It is important to acknowledge that patients adhering to the Mediterranean diet might engage in other health-promoting behaviors, such as enhanced food literacy and increased physical activity, which can also influence the findings of observational studies [52,53,54].

The dietary and lifestyle changes should be viewed as complementary to current medical interventions. Each diet has individual benefits and disadvantages. The consumption of minimally processed, whole, plant-based foods is a feature of these healthy diets that is supported by a growing body of studies that highlight potential benefits for people with CKD. In order to improve their health as much as possible, patients should be given the knowledge and resources they need to adopt the diet that is most applicable to their circumstances and stage of CKD [55]. However, various dietary strategies of recent years and the many new discoveries still require careful attention and additional studies.

### 3.3. Pharmacological Therapies

#### 3.3.1. Mineralocorticoid Receptor Antagonists—Finerenone

Mineralocorticoid Receptor Antagonists (MRAs) are a group of drugs, the best-known representatives of which are spironolactone and eplerenone—classic steroid antagonists. Among the non-steroidal MRAs we can distinguish, for example, finerenone, exaserenone, and apararenone [56]. For example, the non-steroidal MRA finerenone shows a balanced distribution between the heart and kidneys compared to spironolactone or eplerenone, which are concentrated in the kidneys. Another difference is that finerenone has no active metabolites, potentially reducing its long-term effects on sodium–potassium balance, and it shows a shorter half-life [57]. Finerenone also has more potent anti-inflammatory and antifibrotic effects than steroidal mineralocorticoid receptor antagonists [58,59,60]. Its mechanism of action involves blocking excessive activation of the MR (mineralocorticoid receptor) and MR-mediated sodium reabsorption [59,61]. Its effects have been particularly noted in patients with CKD and concomitant T2DM, who are known to be at high risk for cardiovascular morbidity and mortality [62,63]. Finerenone has been shown to reduce albuminuria and N-terminal pro B-type natriuretic peptide (NT-proBNP), and cause a lower risk of hyperkalemia compared to steroidal MRAs [61,64,65]. To evaluate the effects and safety of finerenone, two phase III trials were conducted: FIDELIO-DKD (Finerenone in Reducing Kidney Failure and Disease Progression in Diabetic Kidney Disease; NCT02540993) and FIGARO-DKD (Finerenone in Reducing Cardiovascular Mortality and Morbidity in Diabetic Kidney Disease; NCT02545049) [61,66]. Both of them were double-blind, randomized, and placebo-controlled trials. A comparison of the inclusion criteria is shown in Table 2 below [58,59,66].

The FIDELIO-DKD trial involved patients with T2DM and more advanced CKD whereas FIGARO-DKD involved patients with T2DM in earlier stages of CKD but at high risk of CVD. Randomized patients were administered once a day oral treatment with finerenone or the placebo. In total, these trials included 13,026 patients, among whom 5935 had a history of Atherosclerotic Cardiovascular Disease (ASCVD). The results showed that the drug significantly reduced the risk of cardiovascular and renal disease in patients with T2DM, independently of the presence of ASCVD. The safety profile of finerenone did not differ between patients with and without ASCVD, and overall it was well tolerated [59,63,66]. In July 2021, finerenone was approved by the U.S. Food and Drug Administration (FDA) to reduce the risk of kidney function decline, cardiovascular death, myocardial infarction, kidney failure, and hypertensive heart failure in people associated with T2DM. Finerenone, under the name Kerendia, is the only MRA known to be available for these indications [61,67].

#### 3.3.2. Antibody Therapy—Canakinumab

Canakinumab is a human monoclonal antibody directed against interleukin IL-1β [68,69]. IL-1β, a pro-inflammatory cytokine, is activated by (NOD)-like receptor protein 3 (NLRP3) at the inflammasome in the kidney. This process is thought to correlate with the development of CKD [70,71]. Activation of IL-1 β promotes chronic inflammation, such as atherosclerosis, whereas blockage of IL-1 β is related to cardiovascular (CV) protection [72]. Currently, canakinumab is a drug that has been approved by the U.S. FDA for the treatment of Familial Mediterranean fever (FMF), intermittent hyperimmunoglobin D syndromes, and systemic juvenile idiopathic arthritis (sJIA) [68,69]. However, studies are still underway to test the therapeutic effect of canakinumab in patients with CKD. These studies often include patients with FMA. FMF is an autosomal recessively inherited autoinflammatory disease characterized by paroxysmal attacks of serositis accompanied by fever, which usually lasts 12–72 h, that may result in renal amyloidosis [73,74,75]. The most common drug for treating amyloidosis is colchicine. However, it is ineffective in patients who are intolerant to colchicine, as well as in patients with frequent colchicine-resistant attacks [76]. A randomized trial was conducted that enrolled colchicine-resistant FMF patients. One group was administered canakinumab via the subcutaneous route and the other was given the placebo. After 16 weeks, 61% of patients in the first group presented a complete response to treatment compared to only 6% in the placebo group. Therefore, it can be considered that canakinumab could be a very useful drug in the future for the treatment of CKD with a particular focus on that accompanying FMF. However, the drug still requires clinical trials to be fully assured of its efficacy [76,77].

#### 3.3.3. Pentoxifylline

Pentoxifylline (PTF) methylxanthine derivative is a non-specific phosphodiesterase inhibitor [78,79]. The mechanism involves inhibition of cyclic -3′,5′-phosphodiesterase (PDE), resulting in an increase in the intracellular concentration of cyclic adenosine monophosphate (cAMP) and causing activation of protein kinase A (PKA) [80]. It improves microcirculation and has strong hematopoietic properties [78,81]. An additional advantage is its strong antiproliferative and anti-inflammatory properties [82]. Indications for use include peripheral vascular diseases such as chromaffinism and it has also found use in CKD. Depending on the indication, the dose of oral pentoxifylline is 400 mg two or three times a day orally with a meal [78,79]. A 2012 randomized trial showed that treatment of 91 patients with this drug for one year reduced serum levels of both high-sensitivity C-reactive protein (hs-CRP), tumor necrosis factor α (TNF-α), and fibrinogen, while eGFR increased by 2.4 mL/min/1.73 m² [79]. Other studies have also shown that the use of PTF improves renal function in CKD patients and reduces proteinuria [81]. As for side effects, the most common are dizziness and gastrointestinal symptoms [80]. Tremors and bleeding also occurred, but were much less frequent [82]. These symptoms occurred most frequently when the dose was not adjusted for the degree of renal failure and the accumulation of harmful metabolites. In the Pentoxifylline for Renoprotection in Diabetic Nephropathy (PREDIAN) study, the frequency of adverse reactions related to abdominal complaints was significantly higher than in the placebo group [83]. In conclusion, the drug is well tolerated and is safe for patients [80].

#### 3.3.4. Glucagon-like Peptide-1 Receptor Agonists

Glucagon-like peptide-1 (GLP-1) receptor agonists are a new but already widely used group of antidiabetic drugs. By affecting the GLP-1 receptor, they influence the action of incretin GLP-1, a protein produced by cells in the small intestine. This leads to lower HbA1c levels by stimulating glucose-dependent insulin secretion and reducing glucagon secretion, gastric emptying, and food intake. In CKD terms, GLP-1 receptor agonists help reduce risk factors by: lowering insulin levels, lowering glucose levels, lowering blood pressure, and causing weight loss [84,85]. Moreover, in recent years, their beneficial effects on the kidneys such as reducing urinary albumin excretion, preventing the onset of macroalbuminuria, and slowing the decline in eGFR over time have been noted. However, the mechanism by which this occurs is not fully known [86]. We can distinguish between two types of GLP-1 receptor agonists. The first group includes short-acting GLP-1 receptor agonists, and these are exenatide and lixisenatide. They have a lesser effect on insulin secretion, a more pronounced slowing of gastric emptying, and a stronger effect on postprandial plasma glucose levels than with fasting glucose. The second group includes long-acting GLP-1 receptor agonists; for example, semaglutide and liraglutide. Liraglutide is often considered the best-studied GLP-1 receptor agonist. By increasing insulin secretion and decreasing glucagon secretion, drugs in this group have less effect on gastric emptying. Consequently, they have a stronger effect on fasting glucose concentrations than on postprandial glucose concentrations and also have a greater effect on 24-h glucose profiles [87]. Two randomized, double-blind, placebo-controlled trials were conducted—SUSTAIN 6 (Trial to Evaluate Cardiovascular and Other Long-term Outcomes With Semaglutide in Subjects With Type 2 Diabetes) and LEADER (Liraglutide Effect and Action in Diabetes). Their results showed that annual eGFR reduction was slower with GLP-1 receptor agonists compared to the corresponding placebo in the general population. This result was more pronounced in CKD patients with baseline eGFR < 60 mL/min/1.73 m^2^. Moreover, another analysis from these two studies showed that patients assigned to treatment with these drugs were more likely to achieve a 30% reduction in UACR compared to the placebo, regardless of baseline UACR [88,89]. The KDIGO guidelines recommend the use of GLP-1 receptor agonists in patients with type 2 diabetes and CKD who have not achieved individualized glycemic goals despite the use of metformin and SGLT2i, or who are unable to use these drugs [90].

#### 3.3.5. Sodium–Glucose Cotransporter-2 Inhibitors

SGLT2—sodium–glucose cotransporter-2—inhibitors are one of a group of hypoglycemic drugs that have also recently found use in treating heart failure and limiting the progression of renal complications often referred to as cardiac-renal complications [91,92]. The drugs were approved by the FDA in 2013 with the indication of treating diabetes. The action of the drugs is to block the sglt2 protein, which is actively involved in the reabsorption of glucose from the proximal renal tubules, resulting in an increase in the amount of glucose in the excreted urine-glucosuria and at the same time lowering the blood glucose concentration. The pleiotropic effect of this drug simultaneously causes a decrease in blood uric acid concentration or body weight [92]. A randomized clinical trial involving patients with type 2 diabetes showed that the effect of SGLT2 can slow the decline in GFR and reduce or completely reverse the progression of proteinuria [91]. In addition, the EMPA-REG OUTCOME study; which tested empagliflozin, the DECLARE-TIMI study; which tested dapagliflozin; and the CANVAS study, which tested canagliflozin, showed that these drugs reduce the risk of CKD progression in patients with type 2 diabetes [93]. According to NICE guidelines, dapagliflozin is recommended when:- It is an add-on medication in patients who are taking the maximum tolerated doses of the angiotensin receptor blocker ARB or the angiotensin-converting enzyme inhibitor ACE in an optimized treatment of,—eGFR is between 25 and 75 mL/min/1.73 m^2^ at the start of treatment, -DM2-uACR is 22.6 mg/mmol or higher [94].

#### 3.3.6. Combination of Angiotensin Receptor Blockers (Valsartan) and Neprilysin Inhibitors

Sacubitril/Valsartan, an angiotensin receptor and neprilysin inhibitor, is a first-in-class ARNI that reduces the risk of death from cardiovascular causes or alleviates symptoms of heart failure and reduces hospitalizations for it [95,96]. These drugs improve renal blood flow and GFR, and inhibit renin release and renal tubules by reducing sodium reabsorption [97]. One study, The Prospective Comparison of ARNI with ACEi to Determine Impact on Global Mortality and Morbidity in Heart Failure (PARADIGM-HF), which studied more than 8000 people, was even earlier prized because of its demonstration of remarkable efficacy over 27 months of follow-up for patients taking enalapril [96]. In this study, creatinine levels above 221 μmol/L and hyperkalemia > 6 mmol/L were significantly less common with ARNI compared to enalapril. In addition, it is noteworthy that there was a reduction in GFR decline, which is a real benefit in including this drug in patients with CKD [95]. Another benefit of this drug is the reduction in deaths from cardiovascular causes and hospitalizations for heart failure, both in patients with and without CKD [96,97]. According to the European Society of Cardiology, the drug Is recommended as a replacement for ACEi or ARB in patients with symptomatic HF and LVEF ≤ 35%. This study also showed that there may be a slowdown in the deterioration of kidney function despite a slight increase in albumin, so further research on these drugs is needed [96].

### 3.4. Kidney Replacement Therapy

When pharmacotherapy proves insufficient and KRT is unavoidable, several factors must be considered before commencing treatment, as well as all associated advantages and disadvantages. KRT encompasses in-center hemodialysis, home hemodialysis, and peritoneal dialysis (PD) [98]. In addition to conventional hemodialysis (HD), depending on the patient’s needs, hemodiafiltration (HDF) with pre- and post-dilution, acetate-free biofiltration (AFB), hemofiltration (HF), and expanded HD is used [99]. Dialysis represents a form of renal replacement therapy in which the kidney’s blood filtering function is supplemented by artificial equipment facilitating the removal of surplus water and toxins, thus enabling the body to maintain homeostasis. It stands as the principal treatment for chronic kidney disease in those patients awaiting kidney transplantation, ineligible for the procedure, or unlikely to receive a kidney transplant.

The decision to initiate the dialysis should be founded upon a comprehensive assessment of the patient’s symptoms, laboratory results, and GFR level, in conjunction with the patient’s unique preferences and quality of life considerations. According to the KDIGO guidelines, an eGFR of <20 mL/min per 1.73 m² signifies an appropriate moment to contemplate KRT implementation. Additionally, the presence of at least one of four specific factors warrants consideration for dialysis initiation: manifestation of symptoms or signs directly attributable to renal failure (such as serositis, acid-base or electrolyte disturbances, pruritus), inability to effectively control volume status or blood pressure, progressive decline in nutritional status despite dietary intervention, or cognitive impairment [98]. Other compelling indications encompass encephalopathy, pericarditis, and pleuritis stemming from severe uremia [6]. A comprehensive compilation of main indications for initiating dialysis can be found in Table 3. Notably, guidance regarding the optimal timing for initiating dialysis in children remains lacking. The current focus is on the development of the KDIGO 2023 guidelines, which are poised to incorporate indications for KRT in pediatric cases. These indications will include factors like inadequate growth despite optimized nutrition, growth hormone intervention, and appropriate medical care.

The initiation of dialysis therapy should be implemented at the right time. Gradual transition to dialysis and to sustain residual renal function as long as possible is suggested [14]. A study by Fu et al. [100] did not demonstrate a significant survival advantage associated with the early commencement of dialysis. In comparison to dialysis initiation at an eGFR of 6–7 mL/min per 1.73 m², initiation at an eGFR of 15–16 mL/min per 1.73 m² yielded a mere 5.1% maximum reduction in the 5-year mortality risk, translating to an extended survival of merely 1.6 months over 5 years, at the cost of commencing dialysis 4 years earlier. In children, early dialysis initiation is also not associated with a significant survival benefit [101].

Before deciding on dialysis, the presence of comorbidities should also be taken into account. Due to the fact that each dialyzed patient has individual clinical and biochemical issues, the same dialysis procedure may not be able to meet the needs of every patient on chronic dialysis. Dialysis patients afflicted with congestive heart failure (HF) or pulmonary edema exhibit a diminished survival rate [102]. Conversely, Chandna et al. [103] observed no significant survival disparity among patients over 75 years of age with comorbidities undergoing KRT as compared to those undergoing conservative treatment. It is impossible to clearly assess the impact of HDF on improving outcomes and cardiovascular survival in patients because study results are not consistent on this topic [104,105,106,107]. The beneficial effect of HDF is the reduction in the risk of intradialytic hypotension (IDH), which may result in the risk of vascular access thrombosis, mesenteric ischemia, cardiovascular events, intradialytic clinically significant arrhythmia, hospital admission, and mortality [99]. Moreover, this method is effective for patients with anemia and/or inflammation, because it is associated with better removal of middle-sized uremic toxins, such as β2-microglobulin, and inflammatory mediators [108]. Patients with recurrent cardiovascular instability with episodes of IDH should be dialyzed using AFB or the biofeedback system on blood volume. AFB helps prevent arrhythmias in susceptible patients, and, for those with chronic obstructive pulmonary disease requiring dialysis, it prevents an overload of CO_2_ [99].

Due to the diverse advantages and disadvantages associated with each dialysis method, personalization of dialysis therapies is essential before implementing KRT.

Despite the continuous improvement of dialysis, mortality rates for dialysis patients are still high. A study by Chandrashekar A et al. [109] showed that the main causes of high mortality among hemodialysis patients are sepsis and ischemic heart disease. High rates of cardiovascular morbidity and mortality on patients receiving HD may be also related to the hemodynamic effects caused by rapid ultrafiltration [110].

One of the primary causes of sudden cardiac death (SCD) in ESKD patients are electrolyte imbalances, frequently present in patients on HD. These abnormalities can explain arrhythmic phenomena. However, due to the elevated incidence of SCD in peritoneal dialysis patients, it is crucial to consider other risk factors related to cardiac comorbidities and uremia that may also contribute to sudden mortality [111]. Increased dialysis dose, high frequency of dialysis sessions, and appropriate albumin level correlate with better survival [109].

Upon determining a specific method of KRT and in the absence of medical contraindications, appropriate preparations should be initiated. These encompass preparation of an arteriovenous fistula for hemodialysis, patient training in PD, implantation of a Tenckhoff catheter, and conducting serological tests for hepatitis B and C, and human immunodeficiency virus (HIV) [1]. A critical issue are the side effects resulting from the presence of an arteriovenous fistula or hemodialysis graft. They can lead to dilatation of the left atrium and right ventricle, and thus to HF [112]. Furthermore, hemodialysis may be unsafe in patients with low blood pressure. In such instances, extended night dialysis may be useful to remove excess fluid [102]. Conversely, PD is assessed as a safe, effective, and well-tolerated therapeutic method in patients with CKD and HF [113,114]. Among patients with symptomatic fluid overloading, PD improved symptoms and prevented hospitalizations [102]. However, individuals who have undergone multiple abdominal surgeries, resulting in peritoneal scars, should not be considered as candidates for PD [6].

Patient preferences regarding the choice of medical management should be carefully discussed and respected. The implementation of appropriate procedures aimed at improving the quality of life of dialysis patients holds significant importance. There are many studies on the beneficial effect of physical activity on outcomes in patients undergoing hemodialysis [115,116,117,118] and peritoneal dialysis [117,119]. These studies give hope for improving the physical fitness of these patients, and thus the comfort of their lives, which is disturbed by systematic dialysis. Consequently, further research in this domain remains imperative.

### 3.5. Transplantation

When it comes down to the management of ESRD, there are two methods of treatment, which are dialysis therapy or kidney transplantation. The second one, namely kidney transplantation, appears to be a preferred approach. It not only shows reduced mortality risk in every age group in comparison to patients on dialysis, who were on waiting lists, but also enables a better quality of life. However, in the case of the elderly patient group, the most significant threat is the elevated mortality in the postoperative period of time [120,121]. The gain in terms of extending life expectancy is particularly noticeable in the age group between 20 to 39 with diabetes mellitus. After transplantation they can expect to live approximately 25 years, in comparison to 8 years without this procedure, while receiving dialysis treatment. The majority of patients do not undergo transplantation, until the eGFR will be lower than 15 mL/min/1.73 m^2^ [122]. The transplanted kidney may come from either a living or deceased donor, but, before the operation, the presence of possible contraindications has to be assessed. There are also a few possible complications of the transplantation, among others including: rejection of the organ, thrombus formation, bleeding, infections, cancer, cardiovascular disease, or even stroke. The frequency of occurrence of transplanted kidney failure depending on the origin of the organ has been shown in Table 4 [123].

The kidney can be transplanted in two ways, namely by open kidney transplantation (OKT) or by robot-assisted kidney transplantation (RAKT). Despite the fact that OKT is the dominant method in most centers, the RAKT shows a number of benefits due to use of minimally invasive surgery. These advantages are decreased risk of operating field contamination, reduced postoperative pain, shortening the length of hospital stay, and smaller incision size compared to the OKT method. Moreover, there are no differences in graft or patient survival, as well as no difference in renal functioning at all [124].

Unfortunately, despite its benefits, RAKT is not the most commonly used method for kidney transplantation, mainly due to the significantly higher cost of performing such a procedure compared to OKT [125]. Nevertheless, it is crucial to take into consideration not just the increased cost of the procedure itself, but also the investment required for purchasing the robotic system [126]. Furthermore, it is essential to highlight that RAKT can be performed safely and effectively only when the operator possesses the necessary expertise in both robotic surgery and kidney transplantation [127].

Anyway, regardless of the transplant method, every transplanted kidney’s function should be regularly assessed, starting with a frequency of twice a week in the first month, gradually reducing this frequency during the first year to at least every 3 months for an indefinite period. There are also situations in which it is important to check serum creatinine concentration, especially during widespread immune response due to, for instance, viral infection. If values exceed 20–25% of the baseline level, this should be a warning sign, because general immune response can be the cause of graft rejection [128]. The above-mentioned rejection may be the result of chronic active T-cell mediated rejection (TCMR) or chronic active antibody-mediated rejection (ABMR). They are categorized as two main subtypes of chronic kidney transplant rejection (CKTR). CKTR is marked by a gradual decline in renal graft function that becomes apparent around one year after the operation and is typically associated with increased blood pressure and proteinuria; it usually occurs in patients with scarce immunosuppression or medication noncompliance. Unfortunately, at present, there are no proven immunotherapies to be effective in prevention or treatment of CKTR, especially in ABMR. However, IVIG (intravenous immunoglobulins), plasmapheresis, and glucocorticoids should be used in active antibody-mediated rejection treatment [129,130].

## 4. Conclusions

CKD is a common and progressive condition. Nevertheless, a variety of factors could have an impact on this process, leading to an increased rate of morbidity and mortality. Early detection and classification are pivotal to implementing interventions that can effectively slow down disease progression and minimize associated complications. Further, maintaining kidney function can improve outcomes and can be accomplished through pharmacological interventions, as well as non-pharmacological strategies (such as dietary and lifestyle changes).

In this review, we focused on the important aspects of CKD. Proper diagnosis and its influence on the management of CKD were considered to be of interest. We paid attention to the role of non-pharmacological strategies, such as physical activity and various dietary patterns and their influence on the development of this disease. Moreover, we discussed novel therapies for CKD: finerenone, canakinumab, and pentoxifylline, together with renal replacement therapy. Furthermore, the role of transplantation was concluded.

These findings might shed new light on CKD and prospective targets for prevention and treatment in the future. However, the significant scientific achievements of recent years and the many new discoveries and mechanisms still require careful attention and additional studies.

## Figures and Tables

**Table 1:** Classification of CKD in stages [5].

Stages	GFR Value [mL min/1.73 m^2^]	Classification
I	>90	Normal or High
II	60–89	Slightly decreased
III A	45–59	Mildly to moderately decreased
III B	30–44	Moderately to severely decreased
IV	15–29	Severely decreased
V	<15	Kidney failure

**Table 2:** Comparison of the inclusion criteria in FIDELIO-DKD and FIGARO-DKD [58,59,66].

FIDELIO-DKD	FIGARO-DKD
Age ≥ 18 years	Age ≥ 18 years
Serum potassium ≤ 4.8 mmol/L	Serum potassium ≤ 4.8 mmol/L
Maximum tolerated dose of an RAS inhibitor	Maximum tolerated dose of an RAS inhibitor
T2DM and CKD defined as UACR between 30–300 mg/g, eGFR 25 ≤ 60 mL/min/1.73 m^2^ and diabetic retinopathy or UACR 300–5000 mg/g and eGFR 25 ≤ 75 mL/min/1.73 m^2^	T2DM and CKD defined as UACR between 30–300 mg/g and eGFR 25–90 mL/min/1.73 m^2^ or UACR 300–5000 mg/g and eGFR ≥ 60 mL/min/1.73 m^2^

RAS—renin–angiotensin system; UACR—urine albumin-to-creatinine ratio.

**Table 3:** Main indications for dialysis initiation.

Indication for Dialysis Initiation
eGFR < 20 mL/min per 1.73 m²
symptoms of renal failure (e.g., serositis, acid-base or electrolyte disturbances, pruritus)
inability to control volume status or blood pressure
progressive decline in nutritional status despite dietary intervention
cognitive impairment
signs of uremia (e.g., encephalopathy, pericarditis, pleuritis)

eGFR—estimated glomerular filtration rate.

**Table 4:** The kidney failure rates after transplantation, depending on the type of donor [123].

Origin of the Organ	Deceased Donor	Living Donor
Kidney failure rates within 1 year	4%	3%
Kidney failure rates within 5 years	21%	14%

## Abbreviations

CKD	Chronic kidney disease
GFR	Glomerular filtration rate
NSAIDs	Nonsteroidal anti-inflammatory drugs
RAS	Renin–angiotensin system
AVP	Arginine vasopressin
ESRD	End-stage renal disease
KRT	Kidney replacement therapy
PA	Physical activity
BP	Blood pressure
T2DM	Type 2 diabetes mellitus
IR	Insulin resistance
e-GFR	Estimated glomerular filtration rate
CVD	Cardiovascular disease
CNS	Central nervous system
KD	Ketogenic diets
PKD	Polycystic kidney disease if Intermittent fasting
PBD	Plant-based diets
MRAs	Mineralocorticoid receptor antagonist
NT-proBNP	N-terminal pro B-type natriuretic peptide
FIDELIO-DKD	Finerenone in Reducing Kidney Failure and Disease Progression in Diabetic Kidney Disease
FIGARO-DKD	Finerenone in Reducing Cardiovascular Mortality and Morbidity in Diabetic Kidney Disease
MR	Mineralocorticoid receptor
UACR	Urine albumin-to-creatinine ratio
ASCVD	Atherosclerotic cardiovascular disease
CV	Cardiovascular
FDA	Food and Drug Administration
IL-1β	Interleukin 1β
NLRP3	(NOD)-like receptor protein 3
FMF	Familial Mediterranean fever
sJIA	Systemic juvenile idiopathic arthritis
PDE	Phosphodiesterase
cAMP	Cyclic adenosine monophosphate
PKA	Protein kinase A
hs-CRP	High-sensitivity C-reactive protein
TNF-α	Tumor necrosis factor *α*
PTF	Pentoxifylline
KDIGO	Kidney Disease: Improving Global Outcomes
PD	Peritoneal dialysis
HD	Hemodialysis
HDF	Hemodiafiltration
AFB	Acetate-free biofiltration
HF	Hemofiltration
HF	Heart failure
HIV	Human immunodeficiency virus
IDH	Intradialytic hypotension
SCD	Sudden cardiac death
OKT	Open kidney transplantation
RAKT	Robot-assisted kidney transplantation
TCMR	T-cell mediated rejection
ABMR	Antibody-mediated rejection
CKTR	Chronic kidney transplant rejection
IVIG	Intravenous immunoglobulins
PREDIAN	Pentoxifylline for Renoprotection in Diabetic Nephropathy
LEADER	Liraglutide Effect and Action in Diabetes
ARNI	Angiotensin receptor-neprilysin inhibitor
LVEF	Left ventricle ejection fraction
ACEI	Angiotensin-converting enzyme inhibitor
ARB	Angiotensin II receptor blocker
NICE	National Institute for Health and Care Excellence
